# One Year Overview and Follow-Up in a Post-COVID Consultation of Critically Ill Patients

**DOI:** 10.3389/fmed.2022.897990

**Published:** 2022-07-14

**Authors:** Jessica González, María Zuil, Iván D. Benítez, David de Gonzalo-Calvo, María Aguilar, Sally Santisteve, Rafaela Vaca, Olga Minguez, Faty Seck, Gerard Torres, Jordi de Batlle, Silvia Gómez, Silvia Barril, Anna Moncusí-Moix, Aida Monge, Clara Gort-Paniello, Ricard Ferrer, Adrián Ceccato, Laia Fernández, Ana Motos, Jordi Riera, Rosario Menéndez, Darío Garcia-Gasulla, Oscar Peñuelas, Gonzalo Labarca, Jesús Caballero, Carme Barberà, Antoni Torres, Ferran Barbé

**Affiliations:** ^1^Department of Pulmonary, Hospital Universitari Arnau de Vilanova and Santa Maria, Lleida, Spain; ^2^Translational Research in Respiratory Medicine Group, Lleida, Spain; ^3^Lleida Biomedical Research Institute, Lleida, Spain; ^4^Centro de Investigación Biomédica en Red (CIBER) of Respiratory Diseases, Institute of Health Carlos III, Madrid, Spain; ^5^Intensive Care Department, Vall d’Hebron Hospital Universitari, Shock, Organ Dysfunction and Resuscitation (SODIR) Research Group, Vall d’Hebron Institut de Recerca, Barcelona, Spain; ^6^Department of Pulmonary, Hospital Clinic, Universitat de Barcelona, Institut d’Investigacions Biomèdiques August Pi i Sunyer (IDIBAPS), Barcelona, Spain; ^7^Department of Pulmonary, University and Polytechnic Hospital La Fe, Valencia, Spain; ^8^Barcelona Supercomputing Center, Barcelona, Spain; ^9^Hospital Universitario de Getafe, Madrid, Spain; ^10^Faculty of Medicine, University of Concepción, Concepción, Chile; ^11^Department of Clinical Biochemistry and Immunology, Faculty of Pharmacy, Concepción, Chile; ^12^Intensive Care Department, Hospital Universitari Arnau de Vilanova de Lleida, Lleida, Spain; ^13^Intensive Care Department, Hospital Universitari Santa Maria de Lleida, Lleida, Spain

**Keywords:** COVID-19, CT abnormalities, intensive care unit (ICU), lung function, SARS, SARS-CoV-2, post-COVID syndrome, sequelae

## Abstract

The long-term clinical management and evolution of a cohort of critical COVID-19 survivors have not been described in detail. We report a prospective observational study of COVID-19 patients admitted to the ICU between March and August 2020. The follow-up in a post-COVID consultation comprised symptoms, pulmonary function tests, the 6-minute walking test (6MWT), and chest computed tomography (CT). Additionally, questionnaires to evaluate the prevalence of post-COVID-19 syndrome were administered at 1 year. A total of 181 patients were admitted to the ICU during the study period. They were middle-aged (median [IQR] of 61 [52;67]) and male (66.9%), with a median ICU stay of 9 (5–24.2) days. 20% died in the hospital, and 39 were not able to be included. A cohort of 105 patients initiated the follow-up. At 1 year, 32.2% persisted with respiratory alterations and needed to continue the follow-up. Ten percent still had moderate/severe lung diffusion (DLCO) involvement (<60%), and 53.7% had a fibrotic pattern on CT. Moreover, patients had a mean (SD) number of symptoms of 5.7 ± 4.6, and 61.3% met the criteria for post-COVID syndrome at 1 year. During the follow-up, 46 patients were discharged, and 16 were transferred to other consultations. Other conditions, such as emphysema (21.6%), COPD (8.2%), severe neurocognitive disorders (4.1%), and lung cancer (1%) were identified. A high use of health care resources is observed in the first year. In conclusion, one-third of critically ill COVID-19 patients need to continue follow-up beyond 1 year, due to abnormalities on DLCO, chest CT, or persistent symptoms.

## Introduction

Since the beginning of the severe acute respiratory syndrome coronavirus 2 (SARS-CoV-2) infection in December 2019, more than 300 million COVID-19 cases have been confirmed globally, and more than 5.7 million people have died (1). A far from negligible proportion of hospitalized patients (20–67%) may develop a more severe disease resulting in acute respiratory distress syndrome (ARDS) (2, 3). This has generated a surge of patients who require respiratory support with invasive or non-invasive mechanical ventilation (IMV and NIMV) (3, 4), overburdening ICUs worldwide.

COVID-19 continues to be a public health emergency of international concern due to the enormous global disease burden. As a result of this situation, there is growing interest in the long-term sequelae after recovery from acute COVID-19. Previous reports indicate that at 6 months of follow-up, at least three-quarters of COVID-19 survivors discharged from the hospital still had persisting symptoms (5–7). Importantly, patients with more severe acute disease and those who were critically ill during their hospital stay had a higher risk of lung diffusion impairment (up to 56%) and radiological abnormalities (4, 6). To date, the literature on 1-year outcomes after hospital discharge is diverse (8, 9) and has not focused on critically ill COVID-19 survivors. Specifically, a study published recently (10) found that those who were critically ill during the hospital stay presented more pulmonary damage on chest CT (87%) and lung diffusing impairment (54%) at the 12-month follow-up.

In this respect, we aimed to describe what happens to the patients who needed ICU admission due to COVID-19 infection 1 year after their hospital discharge. We deeply describe the clinical follow-up, which includes an evaluation of symptoms, respiratory assessment (including lung volumes, DLCO, and 6-minute walking test) and a chest CT scan 3, 6, and 12 months after hospital discharge. Moreover, a questionnaire to evaluate persistent symptoms and post-COVID syndrome was performed at 1 year of follow-up in all patients.

## Materials and Methods

### Study Design and Population

This was a prospective observational study performed in patients who had a critical care admission due to COVID-19 between March and August 2020 in Hospital Universitari Arnau de Vilanova and Hospital Universitari Santa Maria in Lleida (Spain). The study is a subset of the ongoing multicenter study CIBERESUCICOVID (NCT04457505) and follows the Strengthening the Reporting of Observational Studies (STROBE) statement.

The study was approved by the Medical Ethics Committee (CEIC/2273). Informed consent was acquired (written and/or verbal) from all patients.

The main objective of this study was to describe the following at 1 year after a critical COVID-19 infection: (1) a general perspective of these patients, (2) the follow-up of the survivors in the context of a clinical post-COVID unit, and (3) the prevalence of post-COVID syndrome in these patients.

### Inclusion and Exclusion Criteria

All patients were positive for SARS-CoV-2, were older than 18 years and had been admitted to the ICU. Follow-up of patients who survived was based on the following exclusion criteria: (i) treatment with palliative care, (ii) follow-up in another center, and (iii) severe mental disability that made it impossible to assess pulmonary function.

### Clinical Data Collection

#### Clinical Data During Hospital Stay

Patient sociodemographic and comorbidity data and clinical, vital, ventilator, and laboratory parameters were recorded at the hospital and ICU admission. We also collected data on the length of ICU and hospital stays, the duration of mechanical ventilation and the need for and duration of prone positioning, treatments received, complications during hospitalization and death.

#### Follow-Up Visit in the Post-COVID Unit

Patients were evaluated at 3, 6, and 12 months after hospital discharge. General and respiratory symptoms, as well as quality of life and anxiety and depression, were assessed as previously described (11). The protocols for the pulmonary function tests, 6-minute walking test and chest CT scan of the thorax were also previously described (9).

The post-COVID unit is a consultation based on the joint evaluation of a pulmonologist (JG), two nurses (MA, SS), and a physiotherapist (AM) with experience in the management of post-COVID and chronic respiratory patients. Patients were discharged when they had clinically recovered from pulmonary damage due to COVID-19. Nevertheless, many others were referred to other consultations due to previous existing pulmonary conditions (such as COPD or emphysema) or other comorbidities (neurological, cardiological, etc.).

#### Post-COVID Syndrome

We aimed to describe post-COVID syndrome prevalence after 12 months of hospital discharge in all critical COVID-19 survivors. There have been several definitions of this condition proposed in the last year (12). A recent study supported by the World Health Organization (WHO) (13) suggested post-acute COVID-19 as the presence of symptoms such as fatigue, shortness of breath, and cognitive dysfunction that impact daily quality of life after 3 months of probable or confirmed SARS-CoV-2 infection, which are not explained by other alternative diagnoses. Symptoms might be persistent or new onset within at least 2 months.

We evaluated these domains (fatigue, shortness of breath, and cognitive dysfunction) by using standardized and validated questionnaires. The Functional Assessment of Chronic Illness Therapy (FACIT) is a questionnaire that assesses self-conception of fatigue and its impact on health-related quality of life in the last 7 days. It contains 13 items from 0 (not very fatigued) to 4 (very fatigued), where a higher score indicates a better quality of life (14, 15). The British Columbia Cognitive Complaints Inventory (BC-CCI) is a 6-item scale that measures perceived cognitive impairments such as problems with concentration, memory, expressing thoughts, word finding, slow thinking, and difficulty solving problems in the past 7 days (16). A higher score reveals more severe cognitive complaints (17). Finally, we used the modified Medical Research Council (mMRC) scale to define the presence of dyspnea in routine clinical practice.

#### Statistical Analysis

Descriptive statistics of the mean (standard deviation) and median (25th percentile; 75th percentile) were estimated for quantitative variables with normal and non-normal distributions, respectively. The absolute and relative frequencies were calculated for qualitative variables. Relative frequencies were calculated excluding missing data. Categorical variables were compared using the chi-squared test or Fisher’s exact test, whereas continuous variables were compared using the non-parametric Mann–Whitney *U* test or *t*-test, depending on whether the variable was normally distributed (Shapiro Wilk test). The *p*-value for the trend was computed from the Pearson test when the variable was normal and from the Spearman test when it was continuous non-normally distributed. For categorical variables, the p value for the trend was computed from the Mantel–Haenszel test. The p value threshold defining statistical significance in all analyses was set at 0.05. Data management and statistical analyses were performed using R (version 4.0.2; R Foundation for Statistical Computing) (18).

## Results

### General Description of Hospital Stay

A total of 181 patients were admitted to the ICU due to COVID-19 between March and August 2020. Briefly, they were predominantly middle-aged (median [IQR] 61 [52–67] years old) males (66.9%) with obesity, hypertension and diabetes mellitus as the most frequent comorbidities. Of the total cohort, 37 (20.4%) patients did not survive hospital stay. As expected, the non-survivors showed higher comorbidity, were more severe at ICU admission and presented more frequently with acute renal failure than survivors (Table 1; Supplementary Table 1).

**TABLE 1:** Baseline characteristics.

	ALL	Survivors	Non-survivors		
	*n* = 181	*n* = 144	*n* = 37	*P*-value	*n*
	Median [IQR], mean (sd) or n (%)	Median [IQR], mean (sd) or n (%)	Median [IQR], mean (sd) or n (%)		
**Sociodemographic data**					
Age, *years*	61.0 [52.0;67.0]	60.0 [48.0;66.0]	67.0 [62.0;73.0]	**<0.001**	181
Sex, *female*	60 (33.1%)	51 (35.4%)	9 (24.3%)	0.279	181
Smoking history				0.038	181
*Non-smoker*	90 (49.7%)	74 (51.4%)	16 (43.2%)		
*Former*	57 (31.5%)	49 (34.0%)	8 (21.6%)		
*Current*	12 (6.63%)	7 (4.86%)	5 (13.5%)		
*Unknown*	22 (12.2%)	14 (9.72%)	8 (21.6%)		
Time from symptoms to hospital admission, *days*	7.00 [5.00;9.00]	7.00 [5.00;9.00]	6.00 [4.00;8.00]	0.336	180
Time from symptoms to ICU admission, *days*	8.00 [6.00;11.0]	8.00 [7.00;11.0]	8.00 [5.00;11.0]	0.678	180
**Comorbidities**					
Obesity	81 (45.5%)	60 (42.6%)	21 (56.8%)	0.174	178
Hypertension	78 (43.1%)	58 (40.3%)	20 (54.1%)	0.186	181
Diabetes mellitus (Type I/II)	42 (23.2%)	25 (17.4%)	17 (45.9%)	**0.001**	181
Chronic heart disease	22 (12.2%)	13 (9.03%)	9 (24.3%)	**0.021**	181
COPD/Bronchiectasis	14 (7.73%)	9 (6.25%)	5 (13.5%)	0.166	181
Chronic renal disease	11 (6.08%)	6 (4.17%)	5 (13.5%)	**0.049**	181
Asthma	10 (5.52%)	10 (6.94%)	0 (0.00%)	0.218	181
HIV	2 (1.10%)	1 (0.69%)	1 (2.70%)	0.368	181
Immunological disorders	1 (0.55%)	0 (0.00%)	1 (2.70%)	0.204	181

*IQR, interquartile range [p25;p75]; sd, standard deviation; HIV, human immunodeficiency virus. Bold values are statistically significant p-values.*

Focusing on the survivors, the median (IQR) ICU stay was 9 (5–24.2) days, and the overall hospitalization duration was 22 (13–37) days. During the ICU stay, 50.7% of patients required IMV with a median (IQR) duration of 17 (10–25) days. Prone positioning was needed in 47.2% of the patients. Patients were mostly treated with corticosteroids (79.2%), hydroxychloroquine (59.7%), lopinavir/ritonavir (56.9%), tocilizumab (49.3%), and remdesivir (25.0%). Moreover, 95.8% of patients received thromboprophylaxis therapy, and 96.5% received antibiotic therapy. The most frequent complications were septic shock (25.7%) and acute renal failure (16.7%) (Table 1; Supplementary Table 1).

### Post-COVID Unit: Clinical Follow-Up

**FIGURE 1:**
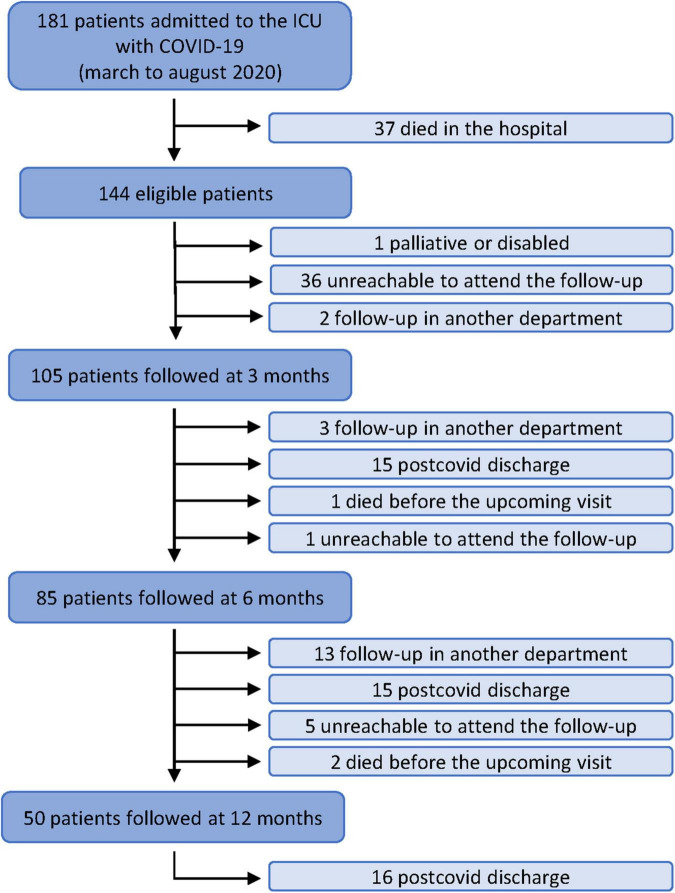
Flowchart of the study.

Figure 1 shows the flowchart of the study and the clinical management during the clinical consultation. After hospital discharge, of the 144 eligible patients, 36 were unreachable or decided not to participate in the follow-up, one was severely disabled, and two underwent follow-up in another center. This left 105 patients who started the clinical follow-up in the post-COVID unit at 3 months after hospital discharge. Patients who did not attend the follow-up visit showed similar sociodemographic and clinical characteristics (except smoking habit and hospital duration) to the patients who did attend the consultation (Supplementary Table 2).

#### Three-Month Follow-Up

Of the 105 patients, 97 and 93 were able to perform pulmonary function tests and 6MWTs, respectively (Table 2). At this point, the proportions of patients with abnormal TLC and DLCO were 38.6 and 82%, respectively. In general, the patients had exercise test results that were lower than expected values (19) with a mean (SD) percent predicted 6-minute walk distance (PP-6MWD) of 83.7% (26) and an average oxygen saturation of 95.3% (1.98). The CT scans showed a high proportion of lung affectation, most frequently with ground-glass opacities (56.6%), followed by mixed ground-glass opacities (29.3%) and consolidation (17.2%). Forty-three (43.4%) and 28 (28.3%) patients showed reticular and fibrotic patterns, respectively, and the mean (SD) of pulmonary lobes affected by ground-glass or consolidation was 3.0 (2.0) with a mean (SD) TSS of 5.8 (4.6) (Table 2).

**TABLE 2:** Description of pulmonary function, 6MWT, and chest CT findings of patients followed at 3, 6, and 12 months.

	Three months	Six months	Twelve months	
	Mean (sd) or n (%)	Mean (sd) or n (%)	Mean (sd) or n (%)	*p* for trend
**Post-COVID consultation discharge**	*n* = 104	*n* = 105	*n* = 105	**<0.001**
Exitus	0 (0.00%)	1 (0.95%)	3 (2.86%)	
None	87 (83.7%)	57 (54.3%)	32 (30.5%)	
Loss to follow-up	0 (0.00%)	1 (0.95%)	6 (5.71%)	
Yes	17 (16.3%)	46 (43.8%)	64 (61.0%)	
**Pulmonary function**				
FVC, %	*n* = 97	*n* = 78	*n* = 38	
	78.1 (15.5)	79.8 (14.7)	86.5 (16.8)	**0.009**
FEV1, %	*n* = 96	*n* = 78	*n* = 38	
	86.0 (17.4)	87.1 (16.5)	91.2 (17.7)	0.138
FEV1 to FVC ratio (categorical)	*n* = 95	*n* = 77	*n* = 37	0.556
≥70%	92 (96.8%)	74 (96.1%)	35 (94.6%)	
<70%	3 (3.16%)	3 (3.90%)	2 (5.41%)	
TLC, %	*n* = 96		*n* = 22	
	82.9 (18.6)	86.3 (18.5)	84.5 (15.6)	0.404
TLC, % (categorical)	*n* = 96	*n* = 70	*n* = 22	0.679
≥80%	59 (61.5%)	48 (68.6%)	13 (59.1%)	
≤50–80%	33 (34.4%)	20 (28.6%)	9 (40.9%)	
<50%	4 (4.17%)	2 (2.86%)	0 (0.00%)	
RV, %	*n* = 96	*n* = 69	*n* = 22	
	90.2 (42.1)	88.3 (34.5)	88.8 (29.5)	0.793
DLCO, *mL/min/mmHg*	*n* = 94	*n* = 79	*n* = 37	
	67.6 (14.7)	65.6 (13.3)	70.6 (13.9)	0.508
DLCO, *mL/min/mmHg* (categorical)	*n* = 94	*n* = 79	*n* = 37	0.553
≥80%	17 (18.1%)	13 (16.5%)	11 (29.7%)	
≤60–80%	51 (54.3%)	36 (45.6%)	17 (45.9%)	
<60%	26 (27.7%)	30 (38.0%)	9 (24.3%)	
**Six-minute walking test**				
PP-6MWD*, %	*n* = 93	*n* = 77	*n* = 37	
	83.7 (26.0)	91.4 (19.9)	95.3 (21.4)	**0.005**
Oxygen saturation, %	*n* = 95	*n* = 77	*n* = 38	
Initial	96.5 (1.26)	96.6 (1.32)	96.7 (1.10)	0.414
Final	95.1 (2.57)	95.1 (2.87)	95.1 (1.62)	0.941
Minimal	94.1 (2.71)	94.3 (2.89)	94.3 (2.15)	0.516
Average	95.3 (1.98)	95.6 (1.87)	95.5 (1.37)	0.374
**Chest CT scan findings**				
Density	*n* = 99	*n* = 81	*n* = 41	
Ground-glass	56 (56.6%)	32 (39.5%)	20 (48.8%)	0.171
Mixed ground-glass	29 (29.3%)	33 (40.7%)	27 (65.9%)	**<0.001**
Consolidation	17 (17.2%)	12 (14.8%)	3 (7.32%)	0.155
Internal structures	*n* = 99	*n* = 81	*n* = 41	
Interlobular septal thickening	81 (81.8%)	62 (76.5%)	41 (100%)	**0.047**
Bronchiectasis	76 (76.8%)	65 (80.2%)	37 (90.2%)	0.082
Atelectasis	22 (22.2%)	17 (21.0%)	11 (26.8%)	0.651
Solid nodule	31 (31.3%)	32 (39.5%)	18 (43.9%)	0.126
Non-solid nodule	2 (2.02%)	6 (7.41%)	0 (0.00%)	0.962
Lesions	*n* = 99	*n* = 81	*n* = 41	0.989
Fibrotic	28 (28.3%)	25 (30.9%)	15 (36.6%)	
None	28 (28.3%)	22 (27.2%)	4 (9.76%)	
Reticular	43 (43.4%)	34 (42.0%)	22 (53.7%)	
No. of lobes affected by ground-glass	*n* = 98	*n* = 81	*n* = 41	
or consolidative opacities	3.06 (2.02)	2.62 (1.95)	3.56 (1.43)	0.443
Total severity score	*n* = 99	*n* = 81	*n* = 41	
	5.88 (4.60)	4.48 (3.68)	4.63 (2.26)	**0.033**

sd, standard deviation; FVC, forced vital capacity; FEV, forced expiratory volume; DLCO, diffusion capacity of the lungs for carbon monoxide; TLC, total lung capacity; RV, residual volume; PP-6MWD, percent predicted 6-minute walk distance. *The PP-6MWD was calculated from standardized prediction equations using the formula PP-6MWD = 6MWD/Predicted 6MWD × 100. Bold values are statistically significant p-values.

**FIGURE 2:**
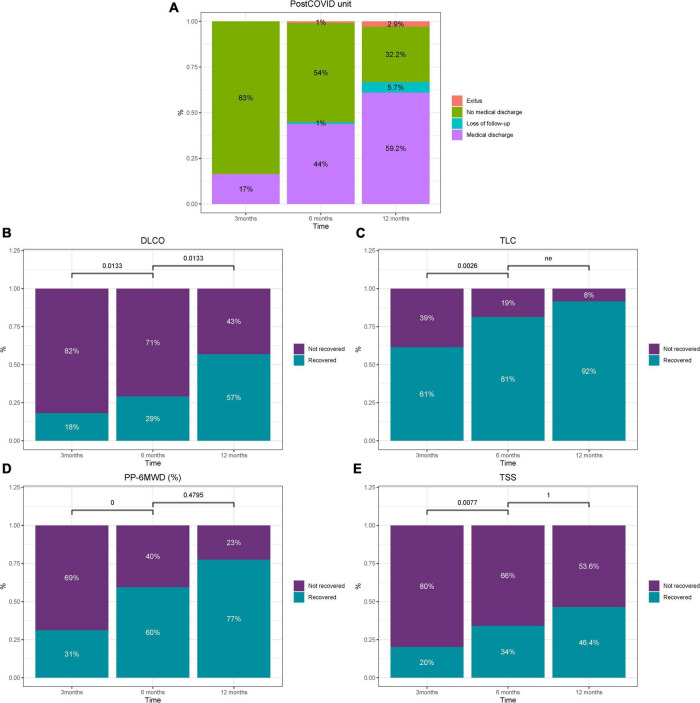
Overview of clinical decisions and percentage of recovered patients regarding post-COVID unit **(A)**, DLCO **(B)**, TLC **(C)**, 6MWT **(D)**, and TSS **(E)** over time. *P*-values were computed using McNemar’s test. ne, not estimable.

After the clinical and functional evaluations, 15 patients were discharged and another 3 transferred for the following consultations: 2 for virtual pulmonary nodules consultation and 1 for psychiatry consultation (Figure 1). Consequently, 83% of patients required a second follow-up visit in the post-COVID unit (Figure 2A).

#### Six-Month Follow-Up

Before this point, one patient died, and another was unreachable and did not attend the follow-up, so 85 patients were evaluated (Figure 1). Of these followed patients, 79 had available pulmonary function tests, showing proportions of abnormal TLC and DLCO of 31.5 and 83.6%, respectively. The PP-6MWD mean (SD) was 91.4% (19.9). Chest CT showed a slight improvement in some parameters regarding density, type of lesions, and TSS (Table 2).

After the clinical assessment, the clinician decided to discharge 15 patients and to transfer another 13 for different consultations: ten to other pulmonary consultations (five to COPD/emphysema and the rest to asthma/vascular/ventilation/pulmonary nodules and lung cancer fast diagnostic track [FDT] consultations), and three to neurology, hematology, and cardiology (Figure 1).

This meant that two-thirds of the patients (67%) in this consultation needed to continue with follow-up (Figure 2A). Again, this was due to the high proportion of patients who did not recover lung diffusion capacity to within the normal range because of COVID-19 damage (Figure 2B).

#### Twelve-Month Follow-Up

Two patients died before the upcoming visit, and five were unreachable and did not attend the follow-up (Figure 1). This left 50 patients evaluated in the consultation, of which 38 required a pulmonary function test, and 41 also received a chest CT.

Of these patients, 40.9 and 70.2% had abnormal TLC and DLCO values, respectively (Table 2). Forty-three, eight and 23 percent of patients did not recover normal values of DLCO, TLC and distance in the 6MWT, respectively (Figures 2B–D). Of these, nine patients (10% of the initial 105 patients) had moderate/severe affectation of DLCO with values below 60%. The mean (SD) PP-6MWD was 95.3% (21.3). The chest CT of these more affected patients showed a high proportion of abnormalities, with the most frequent finding being interlobular septal thickening (100%) and bronchiectasis (90.2%), with all of this in the context of the presence of reticular and fibrotic patterns in 53.7 and 36.6% of patients, respectively. The number of lobes affected by ground-glass or consolidation remained high (mean [SD] of 3.5 [1.4]) (Table 2). Fifty-three percent of patients had abnormal TSS values at this point (Figure 2E).

The pulmonary function, 6MWT and chest CT scan of these 50 patients at 3, 6, and 12 months are depicted in Supplementary Table 3.

After a careful evaluation, the clinician decided to discharge 16 patients. This decision meant that 32.2% of patients, based on the clinical point of view, needed to continue to be monitored beyond 12 months after hospital discharge due to pulmonary sequelae of critical COVID-19.

### Symptoms Related to Post-COVID Syndrome at 12 Months of Follow-Up

To assess the prevalence of post-COVID syndrome 1 year after hospital discharge, a telephone survey was conducted of all 105 initial patients. Three patients had died, and five patients did not respond, so we finally contacted 97 patients.

Thirty-seven percent of patients suffered from mild/moderate/severe cognitive complaints based on the BC-CCI scale, and 33 and 45% had abnormal values in the fatigue and dyspnea scales, respectively. This results in 61.3% of patients showing at least one altered domain. Additionally, the patients had a mean (SD) number of symptoms of 5.7 (4.6), with the most frequent being reduced fitness (700.1%), concentration and/or memory problems (50.5%), muscle weakness (46.4%), tingling and/or pain in the extremities (43.3%), and erectile dysfunction (38.8%), among many others (Table 3).

**TABLE 3:** Prevalence of persistent symptoms and post-COVID syndrome at the 1-year follow-up.

	Twelve-month follow-up	
	*n* = 97	
	Mean (sd) or n (%)	*n*
**Post-COVID syndrome**		
BC-CCI		96
None or minimal cognitive complaints	60 (62.5%)	
Mild cognitive complaints	19 (19.8%)	
Moderate cognitive complaints	13 (13.5%)	
Severe cognitive complaints	4 (4.17%)	
Total score	3.89 (4.76)	96
FACIT score	36.8 (12.3)	96
Score < 30	32 (33.3%)	
Dyspnea		94
0	51 (54.3%)	
1	31 (33.0%)	
2	9 (9.57%)	
3	3 (3.19%)	
Post-COVID syndrome*	57 (61.3%)	93
**Sequelae symptoms**		
Number of symptoms	5.77 (4.66)	97
Reduced fitness	68 (70.1%)	97
Concentration and/or memory problems	49 (50.5%)	97
Muscle weakness	45 (46.4%)	97
Tingling and/or pain in extremities	42 (43.3%)	97
Erectile dysfunction	26 (38.8%)	67
Sleeping problems	36 (37.1%)	97
Joint complaints	32 (33.0%)	97
Reduced vision	31 (32.0%)	97
Hoarseness	27 (28.1%)	96
Hair loss	26 (26.8%)	97
Smell or taste disorder	26 (26.8%)	97
Changes in menstruation	8 (26.7%)	30
Reduced hearing	24 (24.7%)	97
Headache	21 (21.6%)	97
Dizziness	20 (20.6%)	97
Palpitations	20 (20.6%)	97
Skin rash	17 (17.5%)	97
Sore throat or difficulty swallowing	14 (14.4%)	97
Chest pain	14 (14.4%)	97
Loss of appetite	8 (8.25%)	97
Diarrhea or vomiting	6 (6.19%)	97
**Patient Global Impression of Severity (PGI-S)**		**97**
None	48 (49.5%)	
Mild	14 (14.4%)	
Moderate	22 (22.7%)	
Severe	12 (12.4%)	
Very severe	1 (1.03%)	
**Vaccination**		
COVID-19 vaccination	79 (82.3%)	96
COVID-19 brand names		78
Pfizer	36 (46.2%)	
Moderna	11 (14.1%)	
AstraZeneca	27 (34.6%)	
Janssen	4 (5.13%)	
Administered doses	1.34 (0.48)	79
Time to first vaccination, *days*	317 (95.7)	79
**SF-12**		95
Physical score	45.7 (11.1)	
Mental score	48.1 (13.3)	

*Post-COVID syndrome is defined as alterations in fatigue, cognitive disorders, and/or dyspnea. sd, standard deviation; BC-CCI, British Columbia Cognitive Plain Inventory; FACIT, Functional Assessment of Chronic Illness Therapy; SF-12, 12-Item Short Form Survey.

There were no differences in symptoms, including FACIT, BC-CCI, and mMRC scores, between patients who needed to complete the follow-up in the post-COVID unit vs. discharged patients (Supplementary Table 4). Additionally, no significant correlation was observed between objective respiratory measurements and symptoms. Only the mMRC scale showed a significant correlation with DLCO (*r* = −0.3; *p* = 0.027) and the FACIT score with the 6MWT (*r* = 0.3; *p* = 0.04) and TSS (*r* = 0.3; *p* = 0.04) (Supplementary Figure 1).

### Additional Diagnosis and Health Care Use During the 1-Year Follow-Up

During the follow-up, three patients died (Supplementary Table 5). In the clinical context of this post-COVID unit, many other conditions were diagnosed and treated (Supplementary Table 6). Those other conditions included neurological/cognitive problems, coagulation disorders, cardiological problems, diaphragm elevation, and morbid obesity. More importantly, in one patient, a new diagnosis of pulmonary adenocarcinoma was made, and three had a high level of suspicion of either a new diagnosis or a recurrence of lung cancer. Twenty-one (20%) and eight (7.6%) patients were recently diagnosed with emphysema and spirometric COPD, respectively. After careful clinical evaluation, two patients were recruited and accepted into a randomized clinical trial of antifibrotics in post-COVID-19 patients in another hospital.

The use of the national health system was high (Supplementary Table 7). The mean (SD) number of outpatient clinic visits were 12.4 (9.25), with a mean of 5.8 (4.5) and 2.3 (2.7) phone and emergency department visits, respectively. Thirteen patients (13.4%) needed hospitalization, and one was admitted to the ICU. Thirty-six patients (37.1%) attended a pulmonary rehabilitation program.

## Discussion

Our report describes an overview of critically ill patients due to COVID-19 between March and August 2020 and the clinical follow-up of survivors in a single center post-COVID critical care unit for 1 year. The most relevant findings of this study are: first, 32% of patients needed to continue the follow-up in a post-COVID unit beyond 1 year. A total of 10% of these patients had moderate/severe affectation of DLCO (values below 60%), and chest CT showed a high proportion of fibrotic (53.7%) and reticular (36.5%) patterns. Second, during the follow-up period, other conditions and comorbidities (related or not to COVID-19), such as emphysema, COPD, neurocognitive disorders, and lung cancer, were identified. Third, at the 12-month follow-up, a highly variable number of symptoms and post-COVID syndrome were very common (even in discharged patients). Fourth, a high use of health care resources is observed in the first year.

There are numerous studies regarding pulmonary sequelae after COVID-19 at 12 months (8, 10, 20). These prospective cohorts of patients already point to a high prevalence of pulmonary involvement represented by an abnormal DLCO and many chest CT findings. This is especially important in those with the most severe disease in the acute phase, where 54% of patients have abnormal DLCO values and 87% have at least one abnormal chest CT pattern at 1 year of follow-up (10). However, to date, the literature focusing on the long follow-up of critically ill survivors of COVID-19 is scarce (21). Gamberini et al. (21) described 51.5% of patients with abnormal DLCO, with 70.3% of patients having fibrotic changes on chest CT and 40.5% having ground-glass opacities or consolidation at 1 year. These data are even more worrisome than ours, probably because this group focused on invasively ventilated patients. Further studies are needed to create or validate scores to identify patients at high risk of pulmonary sequelae on chest CT (22).

Although all of these studies assessed pulmonary sequelae after COVID-19 at 12 months (8, 10, 20), none of them provided information about the clinical management and follow-up in a real post-COVID consultation. Our work demonstrates that during follow-up, many comorbidities (related to COVID-19 or not) could be diagnosed and should be managed, such as COPD, emphysema, lung cancer, or other non-respiratory conditions. Moreover, the clinical nature of this consultation allowed us to discriminate COVID-19 respiratory sequelae from previous existing pulmonary conditions (and those not previously diagnosed), such as COPD and emphysema.

Another important issue is persistent symptoms and post-COVID syndrome in critically ill COVID-19 survivors. The literature shows that a wide variety of symptoms and impairment of health-related quality of life at 1 year are very frequent (21). Our results go in line with others that shows a high proportion of ongoing symptoms as well as a substantial new disability and reduced health quality of life in critically ill COVID-19 survivors (23). Moreover, our results show no differences in the prevalence of symptoms or post-COVID syndrome between discharged patients and those who needed to continue the follow-up in the unit. This highlights the need for a more precise definition of post-COVID syndrome (24). In our cohort, symptoms such as dyspnea and fatigue were explained by DLCO and FACYT score measurements, while the other symptoms were not. This result should be interpreted with caution because it could be explained by the overlap of ARDS sequelae (25, 26), the so-called postintensive care syndrome (PICS) (27) and the post-COVID syndrome (28). Interestingly, a study performed by Hodgson and colleagues (29, 30) showed that COVID-19 and non-COVID-19 PICS at 6 months after ICU admission are at least phenotypically similar, with similar post-ICU care. Be that as it may and consequently, these critical survivors have a high consumption rate of health resources (31) that must be managed in an adequate post-COVID care unit.

There are some limitations to our study. First, it is a small cohort from a single city which may reduce the external validity and generalizability of the findings. Second, due to the clinical nature of this consultation, we were not able to describe the pulmonary and functional evaluation of all patients who required a critical COVID-19 admission at 12 months. However, we have described the real clinical practice and the follow-up of these patients in a post-COVID unit.

In conclusion, in a single center post-COVID critical care unit, 32% of patients need to continue follow-up beyond 1 year due to the high proportion of patients with abnormal DLCO and chest CT. Many comorbidities (related to COVID-19 or not) were diagnosed during the follow-up. Finally, persistent symptoms and post-COVID syndrome are very common, which leads to high health care consumption.

## Data Availability Statement

The raw data supporting the conclusions of this article will be made available by the authors, without undue reservation.

## Ethics Statement

The studies involving human participants were reviewed and approved by Medical Ethics Committee (CEIC/2273). The patients/participants provided their written informed consent to participate in this study.

## Author Contributions

JG, MZ, IB, DG-C, GT, JB, SG, SB, RF, AC, LF, AMot, JR, RM, DG-G, OP, GL, JC, CB, AT, and FB contributed to the study concept and design. MA, SS, RV, OM, FS, AM-M, AMon, and CG-P contributed to the data acquisition. JG, MZ, IB, DG-C, AM-M, and CG-P contributed to the data analysis and interpretation. FB was the guarantor of the manuscript, had full access to all of the data in the study and took responsibility for the integrity of the data and the accuracy of the data analysis. All authors contributed to the manuscript draft, critically revised the manuscript for important intellectual content, and approved the final version.

## Conflict of Interest

The authors declare that the research was conducted in the absence of any commercial or financial relationships that could be construed as a potential conflict of interest.

## Publisher’s Note

All claims expressed in this article are solely those of the authors and do not necessarily represent those of their affiliated organizations, or those of the publisher, the editors and the reviewers. Any product that may be evaluated in this article, or claim that may be made by its manufacturer, is not guaranteed or endorsed by the publisher.

## Abbreviations

CT: computed tomography
COVID-19: coronavirus disease 2019
ICU: intensive care unit
ARDS: acute respiratory distress syndrome
SARS-CoV-2: severe acute respiratory syndrome coronavirus 2
6MWT: 6-minute walking test.
