# Supplementary material for: One Year Overview and Follow-Up in a Post-COVID Consultation of Critically Ill Patients
Source: Front Med (Lausanne). 2022 Jul 14;9:897990. doi: 10.3389/fmed.2022.897990 (PMC9329578; doi:10.3389/fmed.2022.897990)
Supplement: Supplementary file 1 [file Data_Sheet_1.docx]

***Online supplement***

**ONE YEAR OVERVIEW AND DESCRIPTION OF FOLLOW-UP IN A POST-COVID CONSULTATION OF CRITICAL PATIENTS WITH COVID-19**

Jessica González^1,2,3,4^, María Zuil^1,2,3,4^, Iván D. Benítez^2,3,4^, David de Gonzalo-Calvo^2,3,4^ , María Aguilar^1,2^ , Sally Santisteve^1,2,3,4^ , Rafaela Vaca^1,2^, Olga Minguez^1,2^, Fatty Seck^1,2^, Gerard Torres^1,2,3,4^, Jordi de Batlle^2,3,4^, Silvia Gómez^1,2,3,4^, Silvia Barril^1,2,3,4^, Anna Moncusí-Moix^2,3,4^, Aida Monge^1,2,3,4^, Clara Gort-Paniello^2,3,4^, Ricard Ferrer^4,5^, Adrián Ceccato^4^, Laia Fernández^4,6^, Ana Motos,^4,6^ Jordi Riera,^4,5^ Rosario Menéndez,^4,7^ Darío Garcia-Gasulla,^8^ Oscar Peñuelas,^4,9^ Gonzalo Labarca,^10^ Jesús Caballero^11^, Carme Barberà^12^, Jesús F. Bermejo-Martin^4,13^, Antoni Torres,^4,6^ Ferran Barbé,^1,2,3,4^ *on behalf of the CIBERESUCICOVID Project (COV20/00110, ISCIII)*

Monitored email address of CIBERESUCICOVID: [marguimbau@ciberes.org](mailto:marguimbau@ciberes.org).

**SUPPLEMENTARY FILES**

Table S1: Additional baseline characteristics

|  | **ALL** | **Survivors** | **Nonsurvivors** |  |  |
| --- | --- | --- | --- | --- | --- |
|  | n = 181 | n = 144 | n = 37 | *p value* | n |
|  | Median [IQR],  mean (sd) or n (%) | Median [IQR],  mean (sd) or n (%) | Median [IQR],  mean (sd) or n (%) |  |  |
| **Hospital data** |  |  |  |  |  |
| Hospitalization, *days* | 19.0 [11.0;36.0] | 22.0 [13.0;37.0] | 14.0 [5.00;24.0] | **0.003** | 181 |
| ***Blood tests*** |  |  |  |  |  |
| Urea nitrogen, *mg/dL* | 37.0 [28.0;53.0] | 34.0 [26.0;48.0] | 53.0 [44.5;68.0] | **<0.001** | 177 |
| Creatinine, *mg/dL* | 0.86 [0.72;1.07] | 0.84 [0.70;0.97] | 1.06 [0.82;1.34] | **0.001** | 180 |
| Lactate dehydrogenase, *U/L* | 869 (273) | 811 (245) | 1243 (59.4) | **0.001** | 15 |
| Ferritin, *ng/mL* | 847 [385;1512] | 847 [361;1737] | 970 [750;1202] | 0.821 | 17 |
| C-Reactive protein, *mg/dL* | 139 [67.1;187] | 133 [67.2;182] | 146 [86.2;220] | 0.310 | 171 |
| White blood cell count, *×10^9^/L* | 7.03 [5.26;9.76] | 7.09 [5.29;9.99] | 6.97 [5.08;8.74] | 0.667 | 180 |
| Hemoglobin, *g/dL* | 13.7 [12.6;14.8] | 13.6 [12.7;14.8] | 13.9 [11.8;14.8] | 0.551 | 179 |
| Platelet count, *×10^9^/L* | 190 [148;237] | 192 [154;244] | 182 [136;219] | 0.115 | 180 |
| Lymphocyte count, *×10^9^/L* | 0.86 [0.64;1.16] | 0.87 [0.65;1.10] | 0.79 [0.50;1.31] | 0.615 | 181 |
| International normalized ratio | 1.17 [1.10;1.25] | 1.16 [1.10;1.25] | 1.20 [1.12;1.27] | 0.387 | 173 |
| D-Dimer, *mg/L* | 328 [229;603] | 328 [228;602] | 353 [232;605] | 0.810 | 133 |
| ***Arterial blood gases*** |  |  |  |  |  |
| pH | 7.45 [7.41;7.48] | 7.45 [7.42;7.49] | 7.44 [7.37;7.48] | 0.343 | 156 |
| Partial pressure of oxygen, *mmHg* | 66.0 [55.0;83.5] | 66.0 [54.8;81.2] | 69.0 [57.5;87.5] | 0.403 | 155 |
| Partial pressure of carbon dioxide, *mmHg* | 34.5 [31.0;39.8] | 34.0 [31.0;39.0] | 35.0 [31.0;42.0] | 0.530 | 154 |
| Oxygen saturation, *%* | 94.4 [91.2;96.6] | 94.4 [91.2;96.5] | 94.2 [91.7;96.7] | 0.787 | 175 |
| **ICU data** |  |  |  |  |  |
| ICU, *days* | 10.0 [5.00;24.0] | 9.00 [5.00;24.2] | 10.0 [4.00;22.0] | 0.684 | 181 |
| APACHE | 15.0 [11.2;19.0] | 14.0 [10.0;17.2] | 18.0 [15.2;22.8] | **<0.001** | 78 |
| ***Blood tests*** |  |  |  |  |  |
| Urea nitrogen, *mg/dL* | 37.5 [28.0;60.2] | 35.0 [25.5;53.0] | 58.0 [44.5;71.0] | **0.001** | 86 |
| Creatinine, *mg/dL* | 0.74 [0.62;0.91] | 0.71 [0.60;0.86] | 1.07 [0.72;1.25] | **0.001** | 90 |
| Lactate dehydrogenase, *U/L* | 860 [684;1010] | 832 [601;942] | 1010 [872;1103] | **0.061** | 43 |
| Ferritin, *ng/mL* | 927 [464;2112] | 785 [464;2098] | 1435 [746;2551] | 0.509 | 33 |
| C-Reactive protein, *mg/dL* | 155 [59.3;209] | 162 [61.8;207] | 143 [49.2;214] | 0.780 | 88 |
| White blood cell count, *×10^9^/L* | 7.98 [5.99;9.96] | 8.17 [5.99;10.2] | 7.84 [5.24;8.87] | 0.322 | 91 |
| Hemoglobin, *g/dL* | 13.1 [11.7;14.5] | 13.2 [12.3;14.5] | 12.7 [11.0;14.3] | 0.330 | 88 |
| Platelet count, *×10^9^/L* | 222 [176;273] | 224 [177;282] | 219 [170;245] | 0.350 | 90 |
| Lymphocyte count, *×10^9^/L* | 0.76 [0.51;1.02] | 0.79 [0.52;1.10] | 0.64 [0.50;0.86] | 0.187 | 90 |
| International normalized ratio | 1.16 [1.07;1.24] | 1.16 [1.10;1.23] | 1.14 [1.03;1.28] | 0.551 | 83 |
| D-Dimer, *mg/L* | 397 [280;574] | 397 [262;636] | 397 [296;445] | 0.792 | 74 |
| ***Arterial blood gases*** |  |  |  |  |  |
| pH | 7.45 [7.41;7.48] | 7.45 [7.41;7.47] | 7.48 [7.42;7.48] | 0.415 | 65 |
| Partial pressure of oxygen, *mmHg* | 62.0 [53.0;81.0] | 66.0 [54.2;80.5] | 56.0 [48.5;82.0] | 0.358 | 65 |
| Partial pressure of carbon dioxide, *mmHg* | 37.0 [33.0;41.0] | 37.0 [33.2;41.8] | 33.0 [30.0;40.5] | 0.220 | 65 |
| Oxygen saturation, *%* | 94.0 [91.0;96.0] | 94.0 [91.0;96.0] | 93.0 [89.3;95.0] | 0.438 | 86 |
| ***Pharmacological treatment*** |  |  |  |  |  |
| Antibiotics | 173 (95.6%) | 139 (96.5%) | 34 (91.9%) | 0.209 | 181 |
| Anticoagulant | 169 (93.4%) | 138 (95.8%) | 31 (83.8%) | **0.018** | 181 |
| Corticosteroids | 146 (80.7%) | 114 (79.2%) | 32 (86.5%) | 0.440 | 181 |
| Hydroxychloroquine | 106 (58.6%) | 86 (59.7%) | 20 (54.1%) | 0.662 | 181 |
| Lopinavir/Ritonavir | 99 (54.7%) | 82 (56.9%) | 17 (45.9%) | 0.311 | 181 |
| Tocilizumab | 87 (48.1%) | 71 (49.3%) | 16 (43.2%) | 0.636 | 181 |
| Remdesivir | 46 (25.4%) | 36 (25.0%) | 10 (27.0%) | 0.967 | 181 |
| Interferon beta | 19 (10.5%) | 16 (11.1%) | 3 (8.11%) | 0.768 | 181 |
| ***Procedures*** |  |  |  |  |  |
| Ventilatory support |  |  |  |  |  |
| High-flow nasal cannula | 127 (70.2%) | 106 (73.6%) | 21 (56.8%) | 0.072 | 181 |
| CPAP/BIPAP | 112 (61.9%) | 87 (60.4%) | 25 (67.6%) | 0.542 | 181 |
| *CPAP/BIPAP, days* | 3.00 [1.00;5.00] | 3.00 [1.00;4.00] | 3.00 [1.00;6.25] | 0.680 | 110 |
| IMV | 100 (55.2%) | 73 (50.7%) | 27 (73.0%) | **0.025** | 181 |
| *IMV, days* | 15.5 [8.00;26.2] | 17.0 [10.0;25.0] | 12.0 [5.00;28.0] | 0.257 | 100 |
| Prone position | 93 (51.4%) | 68 (47.2%) | 25 (67.6%) | **0.043** | 181 |
| **Hospital complications** |  |  |  |  |  |
| ARDS | 174 (96.1%) | 137 (95.1%) | 37 (100%) | 0.347 | 181 |
| Acute renal failure | 43 (23.8%) | 24 (16.7%) | 19 (51.4%) | **<0.001** | 181 |
| Septic shock | 42 (23.2%) | 37 (25.7%) | 5 (13.5%) | 0.178 | 181 |
| Bleeding | 9 (4.97%) | 6 (4.17%) | 3 (8.11%) | 0.392 | 181 |
| Pulmonary embolism | 5 (2.76%) | 4 (2.78%) | 1 (2.70%) | 1.000 | 181 |
| Stroke | 2 (1.10%) | 2 (1.39%) | 0 (0.00%) | 1.000 | 181 |
| Myocarditis/Pericarditis | 2 (1.10%) | 1 (0.69%) | 1 (2.70%) | 0.368 | 181 |
| Myocardial infarction | 1 (0.55%) | 1 (0.69%) | 0 (0.00%) | 1.000 | 181 |
| Myocardial ischemia | 1 (0.55%) | 0 (0.00%) | 1 (2.70%) | 0.204 | 181 |

Abbreviations: IQR, interquartile range [p25;p75]; sd, standard deviation; ARDS, acute respiratory distress syndrome; IMV, invasive mechanical ventilation

**Table S2:** Patient hospital characteristics according to post-COVID unit attendance

|  | **No Post-COVID** | **Post-COVID** |  |  |
| --- | --- | --- | --- | --- |
|  | n = 39 | n = 105 |  |  |
|  | Median [IQR],  mean (sd) or n (%) | Median [IQR],  mean (sd) or n (%) | *P value* | n |
| **Sociodemographic data** |  |  |  |  |
| Age, *years* | 53.0 [43.0;63.5] | 61.0 [50.0;67.0] | 0.050 | 144 |
| Sex, *female* | 17 (43.6%) | 34 (32.4%) | 0.292 | 144 |
| Smoking history |  |  | <0.001 | 144 |
| *Nonsmoker* | 20 (51.3%) | 54 (51.4%) |  |  |
| *Current* | 3 (7.69%) | 4 (3.81%) |  |  |
| *Former* | 4 (10.3%) | 45 (42.9%) |  |  |
| *Unknown* | 12 (30.8%) | 2 (1.90%) |  |  |
| Time from symptoms to hospital admission, *days* | 7.00 [4.50;10.5] | 7.00 [5.00;8.00] | 0.353 | 143 |
| Time from symptoms to ICU admission, *days* | 8.00 [6.00;11.5] | 8.00 [7.00;11.0] | 0.889 | 143 |
| **Comorbidities** |  |  |  |  |
| Obesity | 16 (44.4%) | 44 (41.9%) | 0.944 | 141 |
| Hypertension | 12 (30.8%) | 46 (43.8%) | 0.220 | 144 |
| Diabetes mellitus (Type I/II) | 5 (12.8%) | 20 (19.0%) | 0.529 | 144 |
| Chronic heart disease | 3 (7.69%) | 10 (9.52%) | 1.000 | 144 |
| Chronic renal disease | 2 (5.13%) | 4 (3.81%) | 0.662 | 144 |
| COPD/Bronchiectasis | 1 (2.56%) | 8 (7.62%) | 0.445 | 144 |
| Asthma | 2 (5.13%) | 8 (7.62%) | 0.729 | 144 |
| HIV | 1 (2.56%) | 0 (0.00%) | 0.271 | 144 |
| **Hospital data** |  |  |  |  |
| Hospitalization, *days* | 15.0 [10.0;27.0] | 23.0 [14.0;40.0] | 0.005 | 144 |
| ***Blood tests and arterial blood gases*** |  |  |  |  |
| Urea nitrogen, *mg/dL* | 32.0 [23.5;48.5] | 35.0 [28.0;48.0] | 0.433 | 142 |
| Creatinine, *mg/dL* | 0.84 [0.70;0.97] | 0.82 [0.70;0.97] | 0.866 | 144 |
| Lactate dehydrogenase, *U/L* | 879 (225) | 781 (260) | 0.513 | 13 |
| Ferritin, *ng/mL* | 499 [325;673] | 1183 [373;1853] | 0.324 | 13 |
| C-Reactive protein, *mg/dL* | 134 [44.5;185] | 130 [68.4;180] | 0.959 | 136 |
| White blood cell count, *×10^9^/L* | 8.63 [6.02;11.4] | 6.72 [5.16;9.56] | 0.016 | 143 |
| Hemoglobin, *g/dL* | 13.2 [12.4;13.9] | 13.8 [12.9;14.9] | 0.074 | 142 |
| Platelet count, *×10^9^/L* | 209 [169;270] | 188 [147;237] | 0.110 | 144 |
| Lymphocyte count, *×10^9^/L* | 0.87 [0.65;1.13] | 0.84 [0.65;1.09] | 0.590 | 144 |
| International normalized ratio | 1.17 [1.12;1.24] | 1.16 [1.10;1.25] | 0.933 | 138 |
| D-Dimer, *mg/L* | 368 [252;921] | 308 [200;491] | 0.080 | 106 |
| pH | 7.44 [7.40;7.48] | 7.45 [7.43;7.49] | 0.316 | 125 |
| Partial pressure of oxygen, *mmHg* | 69.0 [57.0;85.0] | 64.0 [53.0;78.5] | 0.154 | 124 |
| Partial pressure of carbon dioxide, *mmHg* | 36.0 [31.0;40.0] | 34.0 [31.0;38.0] | 0.287 | 123 |
| Oxygen saturation, *%* | 94.6 [92.9;97.9] | 94.1 [90.9;96.0] | 0.134 | 141 |

| **ICU data** |  |  |  |  |
| --- | --- | --- | --- | --- |
| ICU, *days* | 8.00 [4.00;11.5] | 13.0 [5.00;27.0] | 0.025 | 144 |
| APACHE | 13.0 (5.34) | 15.5 (5.43) | 0.093 | 56 |
| ***Blood tests and arterial blood gases*** |  |  |  |  |
| Urea nitrogen, *mg/dL* | 36.0 [33.0;42.5] | 34.0 [25.0;54.2] | 0.431 | 67 |
| Creatinine, *mg/dL* | 0.70 [0.65;0.81] | 0.71 [0.60;0.86] | 0.884 | 71 |
| Lactate dehydrogenase, *U/L* | 742 (192) | 852 (319) | 0.230 | 35 |
| Ferritin, *ng/mL* | 641 [382;980] | 921 [464;2192] | 0.380 | 26 |
| C-Reactive protein, *mg/dL* | 114 [37.7;220] | 162 [82.2;207] | 0.788 | 69 |
| White blood cell count, *×10^9^/L* | 8.94 [7.38;12.0] | 8.07 [5.96;9.97] | 0.230 | 72 |
| Hemoglobin, *g/dL* | 14.0 [12.7;15.0] | 13.2 [12.1;14.4] | 0.191 | 69 |
| Platelet count, *×10^9^/L* | 242 [208;343] | 220 [174;269] | 0.220 | 71 |
| Lymphocyte count, *×10^9^/L* | 0.88 [0.73;1.32] | 0.75 [0.50;1.06] | 0.116 | 71 |
| International normalized ratio | 1.05 [1.02;1.11] | 1.17 [1.11;1.24] | 0.033 | 64 |
| D-Dimer, *mg/L* | 383 [281;511] | 397 [266;656] | 0.834 | 57 |
| pH | 7.46 [7.42;7.47] | 7.44 [7.41;7.47] | 0.823 | 54 |
| Partial pressure of oxygen, *mmHg* | 57.0 [47.0;65.2] | 67.0 [55.0;92.8] | 0.147 | 54 |
| Partial pressure of carbon dioxide, *mmHg* | 39.0 [34.2;45.8] | 37.0 [33.0;40.2] | 0.366 | 54 |
| Oxygen saturation, *%* | 91.5 [86.9;94.2] | 94.5 [91.6;96.3] | 0.061 | 69 |
| ***Pharmacological treatments*** |  |  |  |  |
| Hydroxychloroquine | 19 (48.7%) | 67 (63.8%) | 0.147 | 144 |
| Corticosteroids | 33 (84.6%) | 81 (77.1%) | 0.453 | 144 |
| Anticoagulant | 37 (94.9%) | 101 (96.2%) | 0.662 | 144 |
| Antibiotics | 37 (94.9%) | 102 (97.1%) | 0.612 | 144 |
| Lopinavir/Ritonavir | 16 (41.0%) | 66 (62.9%) | 0.031 | 144 |
| Remdesivir | 10 (25.6%) | 26 (24.8%) | 1.000 | 144 |
| Tocilizumab | 23 (59.0%) | 48 (45.7%) | 0.220 | 144 |
| Interferon beta | 2 (5.13%) | 14 (13.3%) | 0.236 | 144 |
| ***Procedures*** |  |  |  |  |
| Ventilatory support |  |  |  |  |
| High-flow nasal cannula | 28 (71.8%) | 78 (74.3%) | 0.929 | 144 |
| CPAP/BIPAP | 25 (64.1%) | 62 (59.0%) | 0.719 | 144 |
| *CPAP/BIPAP, days* | 3.00 [1.00;6.00] | 3.00 [1.00;4.00] | 0.496 | 86 |
| IMV | 16 (41.0%) | 57 (54.3%) | 0.220 | 144 |
| *IMV, days* | 7.50 [6.00;19.8] | 18.0 [11.0;27.0] | 0.015 | 73 |
| Prone position | 14 (35.9%) | 54 (51.4%) | 0.141 | 144 |
| **Hospital complications** |  |  |  |  |
| ARDS | 35 (89.7%) | 102 (97.1%) | 0.086 | 144 |
| Acute renal failure | 4 (10.3%) | 20 (19.0%) | 0.314 | 144 |
| Bleeding | 1 (2.56%) | 5 (4.76%) | 1.000 | 144 |
| Stroke | 1 (2.56%) | 1 (0.95%) | 0.470 | 144 |
| Pulmonary embolism | 0 (0.00%) | 4 (3.81%) | 0.574 | 144 |
| Myocardial infarction | 1 (2.56%) | 0 (0.00%) | 0.271 | 144 |
| Myocarditis/Pericarditis | 0 (0.00%) | 1 (0.95%) | 1.000 | 144 |
| Septic shock | 6 (15.4%) | 31 (29.5%) | 0.131 | 144 |
| Abbreviations: IQR, interquartile range *[p_25_;p_75_]*; sd, standard deviation; HIV, human immunodeficiency viruses; ARDS, acute respiratory distress syndrome; IMV, invasive mechanic ventilation. | | | | |

**Table S3:** Pulmonary function and chest CT findings of nondischarged patients

|  | **Three months** | **Six months** | **Twelve months** |  |  |
| --- | --- | --- | --- | --- | --- |
|  | n = 50 | n = 50 | n = 50 |  |  |
|  | Mean (sd)  or n (%) | Mean (sd) or n (%) | Mean (sd) or n (%) | *p value* | n |
| **SF-12** | n = 43 | n = 48 | n = 48 |  |  |
| Physical score | 41.0 (11.1) | 42.2 (10.00) | 45.7 (11.4) | **0.040** | 139 |
| Mental score | 51.6 (11.4) | 51.8 (10.7) | 50.1 (11.6) | 0.511 | 139 |
| **Pulmonary function** |  |  |  |  |  |
| FVC, % | n = 45 | n = 46 | n = 38 |  |  |
|  | 74.6 (15.1) | 78.3 (14.5) | 86.5 (16.8) | **0.001** | 129 |
| FEV1, % | n = 44 | n = 46 | n = 38 |  |  |
|  | 83.3 (17.5) | 86.5 (16.9) | 91.2 (17.7) | **0.043** | 128 |
| FEV1 to FVC ratio (categorical) | n = 44 | n = 46 | n = 37 |  |  |
| ≥ 70% | 42 (95.5%) | 45 (97.8%) | 35 (94.6%) | 0.875 | 127 |
| < 70% | 2 (4.55%) | 1 (2.17%) | 2 (5.41%) |  |  |
| TLC, % | n = 45 | n = 41 | n = 22 |  |  |
|  | 81.3 (19.7) | 83.7 (13.5) | 84.5 (15.6) | 0.424 | 108 |
| TLC, % (categorical) | n = 45 | n = 41 | n = 22 |  |  |
| ≥ 80% | 24 (53.3%) | 28 (68.3%) | 13 (59.1%) | 0.735 | 108 |
| ≤ 50%-80% | 18 (40.0%) | 12 (29.3%) | 9 (40.9%) |  |  |
| < 50% | 3 (6.67%) | 1 (2.44%) | 0 (0.00%) |  |  |
| RV, % | n = 45 | n = 41 | n = 22 |  |  |
|  | 89.0 (45.9) | 80.5 (23.0) | 88.8 (29.5) | 0.771 | 108 |
| DLCO, *mL/min/mmHg* | n = 44 | n = 46 | n = 37 |  |  |
|  | 63.6 (13.4) | 64.0 (12.0) | 70.6 (13.9) | **0.021** | 127 |
| DLCO, *mL/min/mmHg* (categorical) | n = 44 | n = 46 | n = 37 |  |  |
| ≥ 80% | 5 (11.4%) | 7 (15.2%) | 11 (29.7%) | 0.069 | 127 |
| ≤ 60%-80% | 23 (52.3%) | 18 (39.1%) | 17 (45.9%) |  |  |
| < 60% | 16 (36.4%) | 21 (45.7%) | 9 (24.3%) |  |  |
| **6-minute walking test** |  |  |  |  |  |
| PP-6MWD, % | n = 44 | n = 48 | n = 37 |  |  |
|  | 79.1 (19.1) | 91.0 (21.1) | 95.3 (21.4) | **<0.001** | 129 |
| Oxygen saturation, % | n = 45 | n = 48 | n = 38 |  |  |
| Initial | 96.3 (1.31) | 96.5 (1.40) | 96.7 (1.10) | 0.256 | 131 |
| Final | 94.8 (2.52) | 94.6 (3.24) | 95.1 (1.62) | 0.651 | 131 |
| Minimal | 93.4 (2.60) | 93.8 (3.25) | 94.3 (2.15) | 0.141 | 131 |
| Average | 95.0 (1.78) | 95.2 (2.02) | 95.5 (1.37) | 0.199 | 131 |

| **Chest CT scan findings** | n = 46 | n = 48 | n = 41 |  |  |
| --- | --- | --- | --- | --- | --- |
| Density |  |  |  |  |  |
| Ground-glass | 29 (63.0%) | 19 (39.6%) | 20 (48.8%) | 0.166 | 135 |
| Mixed ground-glass | 22 (47.8%) | 29 (60.4%) | 27 (65.9%) | 0.088 | 135 |
| Consolidation | 12 (26.1%) | 9 (18.8%) | 3 (7.32%) | **0.023** | 135 |
| Internal structures |  |  |  |  |  |
| Interlobular septal thickening | 45 (97.8%) | 45 (93.8%) | 41 (100%) | 0.591 | 135 |
| Bronchiectasis | 42 (91.3%) | 44 (91.7%) | 37 (90.2%) | 0.867 | 135 |
| Atelectasis | 10 (21.7%) | 9 (18.8%) | 11 (26.8%) | 0.587 | 135 |
| Solid nodule | 13 (28.3%) | 19 (39.6%) | 18 (43.9%) | 0.129 | 135 |
| Nonsolid nodule | 0 (0.00%) | 6 (12.5%) | 0 (0.00%) | 0.908 | 135 |
| Lesions |  |  |  | 0.144 | 135 |
| Fibrotic | 24 (52.2%) | 22 (45.8%) | 15 (36.6%) |  |  |
| Reticular | 18 (39.1%) | 21 (43.8%) | 22 (53.7%) |  |  |
| None | 4 (8.70%) | 5 (10.4%) | 4 (9.76%) |  |  |
| No. of lobes affected by ground-glass or consolidative opacities | 4.22 (1.33) | 3.52 (1.52) | 3.56 (1.43) | **0.031** | 135 |
| Total severity score | 8.57 (3.97) | 6.10 (3.41) | 4.63 (2.26) | **<0.001** | 135 |
| Abbreviations: sd, standard deviation; FVC, forced vital capacity; FEV, forced expiratory volume; DLCO, diffusion capacity of the lungs for carbon monoxide; TLC, total lung capacity; RV residual volume; PP-6MWD, percent predicted 6-minute walk distance. *The PP-6MWD was calculated from standardized prediction equations using the following formula: *PP-6MWD = 6MWD/Predicted 6MWD x 100.* | | | | | |

**Table S4:** Symptoms and post-COVID syndrome comparison between discharged patients beginning at 12 months and those who needed to continue the follow-up.

|  | **Medical discharge before 12 months of follow-up** | **Nonmedical discharge at 12 months of follow-up** |  |  |
| --- | --- | --- | --- | --- |
|  | n = 61 | n = 30 | *p value* | n |
|  | n (%) | n (%) |  |  |
| **Post-COVID syndrome** |  |  |  |  |
| BC-CCI |  |  | 0.243 | 90 |
| None or minimal cognitive complaints | 39 (63.9%) | 16 (55.2%) |  |  |
| Mild cognitive complaints | 13 (21.3%) | 6 (20.7%) |  |  |
| Moderate cognitive complaints | 6 (9.84%) | 7 (24.1%) |  |  |
| Severe cognitive complaints | 3 (4.92%) | 0 (0.00%) |  |  |
| Total score | 3.95 (4.77) | 3.93 (4.27) | 0.984 | 90 |
| FACIT score | 35.8 (12.2) | 38.5 (11.9) | 0.316 | 90 |
| Score<30 | 23 (38.3%) | 8 (26.7%) |  |  |
| Dyspnea |  |  | 0.187 | 88 |
| 0 | 33 (55.0%) | 13 (46.4%) |  |  |
| 1 | 22 (36.7%) | 8 (28.6%) |  |  |
| 2 | 4 (6.67%) | 5 (17.9%) |  |  |
| 3 | 1 (1.67%) | 2 (7.14%) |  |  |
| Post-COVID syndrome* | 37 (62.7%) | 19 (67.9%) | 0.819 | 87 |
| **Sequelae symptoms** |  |  |  |  |
| Number of symptoms | 5.90 (4.71) | 5.70 (3.99) | 0.832 | 91 |
| Reduced fitness | 44 (72.1%) | 20 (66.7%) | 0.770 | 91 |
| Concentration and/or memory problems | 34 (55.7%) | 14 (46.7%) | 0.554 | 91 |
| Muscle weakness | 28 (45.9%) | 15 (50.0%) | 0.885 | 91 |
| Tingling and/or pain in extremities | 22 (36.1%) | 18 (60.0%) | 0.053 | 91 |
| Erectile dysfunction | 17 (42.5%) | 8 (38.1%) | 0.953 | 61 |
| Sleeping problems | 23 (37.7%) | 11 (36.7%) | 1.000 | 91 |
| Joint complaints | 20 (32.8%) | 10 (33.3%) | 1.000 | 91 |
| Reduced vision | 18 (29.5%) | 11 (36.7%) | 0.653 | 91 |
| Hoarseness | 20 (33.3%) | 5 (16.7%) | 0.157 | 90 |
| Hair loss | 19 (31.1%) | 6 (20.0%) | 0.384 | 91 |
| Smell or taste disorder | 16 (26.2%) | 9 (30.0%) | 0.897 | 91 |
| Changes in menstruation | 6 (28.6%) | 2 (22.2%) | 1.000 | 30 |
| Reduced hearing | 16 (26.2%) | 7 (23.3%) | 0.966 | 91 |
| Headache | 16 (26.2%) | 4 (13.3%) | 0.260 | 91 |
| Dizziness | 13 (21.3%) | 6 (20.0%) | 1.000 | 91 |
| Palpitations | 12 (19.7%) | 7 (23.3%) | 0.897 | 91 |
| Skin rash | 11 (18.0%) | 5 (16.7%) | 1.000 | 91 |
| Sore throat or difficulty swallowing | 8 (13.1%) | 5 (16.7%) | 0.752 | 91 |
| Chest pain | 9 (14.8%) | 4 (13.3%) | 1.000 | 91 |
| Loss of appetite | 4 (6.56%) | 3 (10.0%) | 0.680 | 91 |
| Diarrhea or vomiting | 4 (6.56%) | 1 (3.33%) | 1.000 | 91 |

| **Patient Global Impression of Severity (PGI-S)** |  |  | 0.814 | 91 |
| --- | --- | --- | --- | --- |
| None | 28 (45.9%) | 15 (50.0%) |  |  |
| Mild | 9 (14.8%) | 5 (16.7%) |  |  |
| Moderate | 16 (26.2%) | 5 (16.7%) |  |  |
| Severe | 7 (11.5%) | 5 (16.7%) |  |  |
| Very severe | 1 (1.64%) | 0 (0.00%) |  |  |
| **Use of health care resources** |  |  |  |  |
| Outpatient clinic visit | 61 (100%) | 30 (100%) | . | 91 |
| Number | 13.3 (10.6) | 12.1 (6.33) | 0.503 | 91 |
| Phone visit | 55 (90.2%) | 24 (80.0%) | 0.200 | 91 |
| Number | 5.73 (4.28) | 6.17 (5.34) | 0.724 | 79 |
| Emergency department visit | 23 (37.7%) | 10 (33.3%) | 0.860 | 91 |
| Number | 2.78 (3.33) | 1.40 (0.70) | 0.069 | 33 |
| Hospitalization | 4 (6.56%) | 6 (20.0%) | 0.075 | 91 |
| Number | 1.25 (0.50) | 1.17 (0.41) | 0.791 | 10 |
| ICU admission | 0 (0.00%) | 1 (3.33%) | 0.330 | 91 |
| Number | . (.) | 1.00 (.) | . | 1 |
| Professional rehabilitation program | 20 (32.8%) | 16 (53.3%) | 0.098 | 91 |
| Number | 1.00 (0.00) | 1.38 (1.02) | 0.164 | 36 |
| **SF-12** |  |  |  |  |
| Physical score | 46.3 (10.3) | 44.5 (12.5) | 0.488 | 88 |
| Mental score | 46.2 (13.6) | 51.3 (11.7) | 0.071 | 88 |
| (*) Post-COVID syndrome is defined as alterations in fatigue, cognitive disorders and/or dyspnea. Abbreviations: sd, standard deviation; BC-CCI, British Columbia Cognitive Complaints Inventory; FACIT, Functional Assessment of Chronic Illness Therapy. Note: eight patients without complete follow-up in the post-COVID unit were excluded from this analysis. | | | | |

**Table S5:** Causes of death after hospital discharge and during follow-up

|  | **12 months** |
| --- | --- |
|  | n = 105 |
|  | n (%) |
| **Cause of death** |  |
| Anoxic encephalopathy | 1 (0.95%) |
| Infective endocarditis | 1 (0.95%) |
| Unknown cause | 1 (0.95%) |

**Table S6:** Comorbidities diagnosed during follow-up

|  | **12-month follow-up** |
| --- | --- |
|  | n = 97 |
|  | n (%) |
| **Comorbidities diagnosed** |  |
| Lung cancer | 1 (1.03%) |
| Highly suspicious pulmonary nodules | 3 (3.09%) |
| Chronic obstructive pulmonary disease | 8 (8.25%) |
| Asthma | 1 (1.03%) |
| Emphysema | 21 (21.65%) |
| Coagulation disorders | 3 (3.09%) |
| Cardiopathy | 1 (1.03%) |
| Neurocognitive disorders | 4 (4.12%) |
| Morbid obesity BMI (>50) | 1 (1.03%) |

**Table S7a:** Consumption of health care resources during the follow-up.

|  | **12-month follow-up** |
| --- | --- |
|  | n = 97 |
|  | n (%) |
| **Use of health care resources** |  |
| Outpatient clinic visit | 97 (100%) |
| Number | 12.4 (9.25) |
| Phone visit | 83 (85.6%) |
| Number | 5.82 (4.54) |
| Emergency department visit | 36 (37.1%) |
| Number | 2.39 (2.76) |
| Hospitalization | 13 (13.4%) |
| Number | 1.54 (0.97) |
| ICU admission | 1 (1.03%) |
| Number | 1.00 (.) |
| Professional rehabilitation program | 36 (37.1%) |
| Number | 1.17 (0.70) |

**Figure S1:** Correlations between symptoms (FACIT and BC-CCI score and mMRC) and pulmonary measurements (DLCO and TSS).
